# Study on the Impact of the Doppler Effect on the Localization and Identification of Moving Lightning Radiation Sources

**DOI:** 10.3390/s26216911

**Published:** 2026-10-31

**Authors:** Caixia Wang, Lin Liu, Jinyuan Guo, Yuexin Zhang, Zhuling Sun, Mingyuan Liu, Hui Yang

**Affiliations:** 1 School of Applied Science, Beijing Information Science and Technology University, Beijing 100192, China; 18603219303@139.com (L.L.); 18311396549@139.com (J.G.); 15690962752@163.com (Y.Z.); 2 State Key Laboratory of Atmospheric Environment and Extreme Meteorology/Laboratory of Middle Atmosphere and Global Environment Observation, Institute of Atmospheric Physics, Chinese Academy of Sciences, Beijing 100029, China; sunzhuling@mail.iap.ac.cn (Z.S.); lium@mail.iap.ac.cn (M.L.); 3 Guangdong Provincial Key Laboratory of Geophysical High-Resolution Imaging Technology Southern University of Science and Technology, Shenzhen 518055, China; yangh@sustech.edu.cn

**Keywords:** Doppler effect, beamforming algorithm, circular planar antenna array, moving lightning radiation source, localization and identification, very high frequency (VHF)

## Abstract

**Highlights:**

A localization and identification method for lightning radiation sources, particularly in the very-high-frequency (VHF) band, was developed based on a beamforming algorithm, and the relationship between the spatial position of radiation sources and the spatial resolution capability of the circular planar array was investigated. The Doppler effect was incorporated into the localization model for moving lightning radiation sources, and simulations were conducted to investigate the mechanisms by which the radiation frequency and frequency difference between the two lightning radiation sources occurring simultaneously affect localization biases.

**What are the main findings?**

- The localization and identification capabilities for dual lightning radiation sources significantly depend on their spatial positions and elevation angles relative to the receiving circular planar array, with array localization accuracy being directly affected by spatial resolution. As the elevation angle increases, the circular planar array’s spatial resolution for dual radiation sources significantly improves.
- The Doppler effect from moving lightning radiation sources causes systematic localization biases that increase with radiation source radiation frequency. Moreover, greater frequency differences between dual-moving radiation sources will exacerbate the influence of the Doppler effect on localization deviations.

**What are the implications of the main findings?**

- The relationship between spatial resolution and elevation angle, propagation distance, and radiation source height is clarified. This can provide theoretical reference for the deployment of planar array observation systems, selection of observation parameters, and identification of radiation sources, and help improve radiation source localization and spatial resolution capabilities.
- The mechanism through which the Doppler effect and radiation source frequency features impact localization biases is elucidated, revealing that motion-induced frequency shifts play a non-negligible role in localizing high-frequency and multi-frequency moving radiation sources. These findings lay an essential theoretical foundation for future localization error corrections, field observational experiments, and fine-scale VHF localization of lightning channel processes.

**Abstract:**

The Doppler effect induced by the motion of lightning radiation sources, referring to electromagnetic radiation sources associated with lightning discharges (especially in the very-high-frequency (VHF) band), affects the propagation and reception of electromagnetic signals, thereby reducing the accuracy of lightning radiation source localization and identification. In this article, to address this issue, based on the Minimum Variance Distortionless Response (MVDR) beamforming algorithm and by introducing the Doppler effect into the localization and identification theory of stationary radiation sources, simulations are performed to comparatively analyze the localization and identification features of both dual-stationary and dual-moving lightning radiation sources. Then, the impact of Doppler shifts on the localization performance of moving lightning radiation sources is investigated. The results show that circular planar array localization accuracy is closely linked to the spatial resolution of the system. The higher the cloud height, the greater the elevation angle, and the higher the spatial resolution. Furthermore, the impact of the Doppler effect on localization accuracy intensifies with higher radiation source frequencies and larger frequency differences between dual radiation sources, leading to greater localization deviations, particularly for high-frequency VHF emissions. This study reveals the mechanism of how the Doppler effect affects moving lightning radiation source localization, providing theoretical support for subsequent field experimental observations and fine-structure research on lightning channel development.

## 1. Introduction

Lightning discharge channels are characterized by strong transient current, intense branching, and rapid three-dimensional (3D) spatial development [1,2]. The associated very-high-frequency (VHF) radiation pulses span nearly all critical stages of the discharge process—including breakdown of air, leader propagation, return stroke, and the M-component process—and serve as a primary information source for reconstructing the 3D structure of lightning channel and elucidating the underlying discharge physics. Ground flashes and their branched channels not only pose substantial threats to critical infrastructure such as power grids, communication systems, and transportation networks, but also provide natural experimental subjects for investigating intra-cloud charge structures, leader propagation mechanisms, and return stroke processes [3,4,5]. Early studies employing the time-of-arrival (TOA) technique established the foundational framework for 3D lightning localization [6,7]. Since then, the continued development of systems including lightning detection and ranging (LDAR) and lightning mapping array (LMA), short-baseline time difference of arrival (TDOA), and broadband interferometers has enabled the spatiotemporal evolution of intra-cloud flashes, ground flashes, and rocket-triggered lightning channels to be captured with progressively higher resolution [8,9,10,11]. Broadband VHF interferometry further exploits phase differences and cross-correlation information to estimate the direction of arrival (DOA), offering distinct advantages in two-dimensional (2D) and 3D source imaging, continuous recording, and the detection of weak radiation sources [12,13,14,15,16,17,18].

In recent years, lightning localization algorithms have advanced considerably, evolving from traditional TOA and interferometric direction-finding to more sophisticated approaches such as waveform cross-correlation, multiple signal classification (MUSIC), time reversal, Estimation of Signal Parameters via Rotational Invariance Techniques (ESPRIT), and machine learning. These developments aim to improve localization accuracy, source capacity, noise immunity, and computational efficiency [19,20,21,22,23,24,25,26,27,28,29,30,31]. In parallel, high-precision systems such as the low-frequency array (LOFAR) have resolved fine-scale structures within positive leaders, initial breakdown regions, and complex branching morphologies. Observations of return stroke leaders, bidirectional leaders, and fast breakdown processes consistently indicate that lightning radiation sources are not stationary, isolated point sources; rather, they undergo continuous motion during channel extension and charge redistribution [32,33,34,35,36,37,38,39]. If array-based receiving models continue to treat such radiation sources as stationary narrowband point sources, the Doppler frequency shift induced by source motion may alter the frequency, phase difference, steering vector matching, and spatial spectrum peaks of the received signals, ultimately degrading the resolution between adjacent or simultaneously active radiation sources.

The existing literature has discussed the impacts of array configurations, signal-to-noise ratio (SNR), elevation angle, baseline length, phase ambiguity, and algorithmic efficiency on localization results [17,26,27,29,40,41]. However, a systematic analysis of how the Doppler effect impacts the localization and identification of two moving lightning radiation sources, as well as the underlying patterns of these effects, remains lacking.

Ground flashes are frequently accompanied by branching of lightning channels, such that radiation sources originating from different altitudes or propagating at different velocities may enter the array observation window at the same time. When the two radiation sources differ in frequency or kinematic state, the Doppler effect can shift the spectral peak positions produced by beamforming algorithms such as Minimum Variance Distortionless Response (MVDR), leading to reduced inter-source resolution, angular estimation biases, or the appearance of spurious peaks. Once such errors propagate into channel reconstruction, they will inevitably distort branch connectivity, source velocity estimates, and the identification of lightning development characteristics. It is therefore necessary to characterize these effects through simulation. Additionally, the beamforming localization algorithm is simple and easy to implement when the number of array elements is large, remains unaffected by the correlation of lightning radiation sources [42], and is well suited for identifying high-frequency sources and performing medium-to-long-range localization measurements [43].

Motivated by these considerations, in this study, based on the beamforming localization algorithm and combined with the Doppler effect theory and the identification theory of two radiation sources, the localization and identification results of two stationary radiation sources and two moving radiation sources are comparatively investigated through Matlab software simulation. The influence of the Doppler effect on the localization and identification of two moving radiation sources is further analyzed and summarized. This research holds both theoretical value and practical significance for conducting refined studies on the physical discharge process of lightning. In Section 2, the Doppler effect, spatial resolution, and the MVDR beamforming algorithm are introduced. In Section 3, simulation results for the localization and identification of two radiation sources are presented, with particular attention given to the influence of source radiation frequency on the localization and identification of moving radiation sources. The relevant issues are discussed in Section 4, and the conclusions are summarized in Section 5.

## 2. Materials and Methods

### 2.1. Doppler Effect Theory of Electromagnetic Waves

When relative motion occurs between a radiation source and the observation array, the received frequency shifts relative to the emitted frequency, a phenomenon known as the Doppler effect. According to the nature of the waves, it can be classified into mechanical and electromagnetic Doppler effects. For the electromagnetic radiation produced by lightning, the relationship between the received frequency $f_{R}$ and the source radiation frequency $f_{s}$ is governed by the following [44]:

$${f_{R}=f}_{s}\sqrt{\frac{c+V}{c-V}}$$

where c represents the speed of light in vacuum, and V denotes the relative radial velocity between the lightning radiation source and the sensor array. By convention, V takes a positive sign when the radiation source and array approach each other along their line-of-sight (LOS) and a negative sign when they recede.

For a lightning radiation source moving at a velocity V, if the angle between the motion vector and the LOS to the array is α, the projected radial velocity is Vcosα. Consequently, the received frequency is given by

$${f_{R}=f}_{s}\sqrt{\frac{c+Vcos\alpha }{c-Vcos\alpha }}$$

Thus, the magnitude of the Doppler frequency shift depends fundamentally on the radiation source velocity, direction of motion, and emission frequency. Under stationary or low-speed scenarios, the frequency shift is negligible, exerting minimal impact on beamforming localization performance. Conversely, in ultra-high-speed scenarios, such as dart leaders and return strokes whose propagation velocities can reach magnitudes of $10^{7}$ to $10^{8}\;m/s$, the resulting Doppler shift becomes prominent and induces non-negligible phase discrepancies that affect localization accuracy.

### 2.2. Theory and Method of Spatial Resolution for Two Lightning Radiation Sources

In multi-source lightning localization, two spatially proximate sources may become indistinguishable, making it necessary to account for the spatial resolution of the localization system. In this section, a simulation model for spatial resolution capability is built based on the Rayleigh criterion. The theoretical formula for two moving lightning radiation sources is derived mathematically, providing the theoretical framework to simulate and analyze its variation patterns in the subsequent sections.

#### 2.2.1. Rayleigh Criterion for the Resolvability of Two Located Sources

When locating two or more moving lightning radiation sources, the azimuth and elevation angles of the two lightning radiation sources can sometimes differ only marginally, making it possible for the positioning system to fail to distinguish between the two radiation sources. Therefore, for multiple-source scenarios, the positioning system’s ability to accurately identify individual sources must also be considered. In this study, a two-lightning-radiation-source scenario is investigated as an example.

The Rayleigh criterion is adopted to evaluate the resolvability of two lightning radiation sources. Originally established in classical optics by Lord Rayleigh to quantify the diffraction limit of circular apertures, the criterion states that two of point sources are just resolvable when the central maximum of one diffraction pattern coincides with the first minimum (null) of the other. In antenna array signal processing, the discrete sensor array behaves as an equivalent spatial receiving aperture, where the synthesized beam pattern corresponds to the optical diffraction pattern. Therefore, the Rayleigh criterion provides an appropriate and rigorous physical benchmark to define the resolution limit of the circular array system in this study. In practice, the full width at half-maximum (FWHM) is commonly used to characterize resolution: a smaller FWHM indicates finer resolution. The spatial resolution of the simulation system in this study is evaluated on this basis.

#### 2.2.2. Spatial Resolution of Two Located Sources

**Figure 1:**
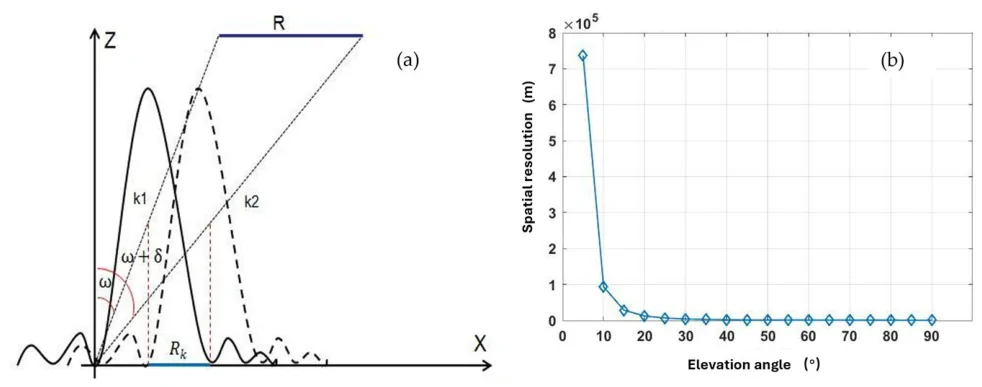
Spatial resolution analysis based on the Rayleigh criterion. Subfigure (**a**): The model of spatial resolution and Rayleigh criterion. Subfigure (**b**): Variation trend of spatial resolution with elevation angle.

Spatial resolution is defined as the minimum angular separation between two equal-intensity signal sources on the lightning source calculation plane that can be accurately identified [45]. In the context of planar array-based beamforming localization, spatial resolution characterizes the ability of the system to distinguish between two closely spaced lightning radiation sources. A spatial resolution model based on the beamforming localization algorithm is constructed in this study, as illustrated in Figure 1a. The XOZ plane in three-dimensional space is selected for the schematic, where **k1** and **k2** denote the wavenumber vectors of two plane waves in the XOZ plane, respectively. The two vectors have equal magnitude, i.e., **|k1| = |k2|** = K. Assuming both plane waves have unit amplitude, the Rayleigh criterion [45] states that the two plane waves are just resolvable when the main-lobe peak of one beam pattern coincides with the first null of the main lobe of the other. The spatial resolution at a given position on the calculation plane therefore equals the main-lobe width of the beam pattern corresponding to a lightning radiation source at that position. Here, δ denotes the angular separation between the two plane waves, ω and ω+δ represent the respective angles of arrival that the incident plane waves make with the Z-axis, with $\theta =90{}^{\circ}-\omega ,K=\sqrt{K_{X}^{2}+K_{Y}^{2}}$ is the magnitude of the projection of K onto the XOY plane, and $R_{k}$ denotes the wavenumber-domain width from the main-lobe peak to the first null.

Based on the Rayleigh resolution criterion, the diffraction-limited angular resolution of an aperture with an effective diameter $D_{eff}$ is approximately ${1.22\;\lambda /D}_{eff}$. Considering the projection of the planar array aperture and the geometrical relationship between the elevation angle and the horizontal source position, the corresponding spatial resolution *R* of a planar array is given by the following [46]:

(1) $$R=\frac{1.22}{{cos}^{3}(90-\theta )}\frac{z_{0}}{D}\lambda =\frac{1.22}{\sin^{3}\theta }\frac{z_{0}}{D}\lambda$$

This depends on the planar array aperture D, the signal frequency $f_{R}$, the angle ω between the source propagation direction and the array axis, and the distance $z_{0}$ from the lightning radiation source calculation plane to the array plane.

#### 2.2.3. Impact of Elevation Angle on the Spatial Resolution of Two Located Sources

Based on the foregoing theory, the spatial resolution of lightning radiation source localization depends on the radiation source propagation direction, which is characterized by both elevation and azimuth angles. For ground flash events, the lightning channel develops predominantly in a combined horizontal and downward direction. Since the azimuth angle variation exhibits spatial symmetry, in this section, the effect of elevation angle on the spatial resolution of two located lightning radiation sources is focused on. Simulations are performed for a range of elevation angles, with the planar array receiving frequency set to $3\times 10^{8}$ Hz and the elevation angle of the incoming wave incremented in steps of 5°. The resulting spatial resolution values are computed and plotted as a function of elevation angle in Figure 1b, where the horizontal axis represents the elevation angle and the vertical axis represents the spatial resolution.

As shown in Figure 1b, spatial resolution improves monotonically with increasing elevation angle θ, with a particularly sharp improvement occurring near $\theta =10^{0}$. The spatial resolution at θ = 60° is 50% higher than at $\theta =0^{\circ }$. This behavior can be explained as follows. At small θ, sinθ in the denominator of Equation (1) can be approximated as θ, so R scales approximately as $\theta^{-3}$, causing the spatial resolution to decrease rapidly with increasing θ. At larger θ, R scales as $\sin^{-3}\theta$, and its rate of change becomes progressively more gradual. This is consistent with the trend in Figure 1b: within the small elevation angle range (0°~10°), spatial resolution is poor and varies rapidly, whereas at larger elevation angles, the resolution is substantially better and changes more smoothly. Accordingly, excessively small elevation angles should be avoided in practice.

### 2.3. Signal Model and MVDR Beamforming Algorithm

#### 2.3.1. Array Signal Model

Considering a lightning electromagnetic radiation source incident on a receiving antenna array consisting of M elements from the elevation angle θ and azimuth direction φ, under the assumption of far-field plane waves, the signal received by the mth element can be expressed as

(2) $$\begin{array}{c} x_{m}\left(t\right)=s(t-\tau_{m}\left(\theta ,\varphi \right))+n_{m}\left(t\right) \end{array}$$

where $s\left(t\right)$ is the transmitted broadband lightning radiation signal, $n_{m}\left(t\right)$ denotes the noise signal, and $\tau_{m}\left(\theta ,\varphi \right)=\frac{r_{m}\cdot k\left(\theta ,\varphi \right)}{c}$ represents the propagation time delay relative to the array coordinate origin, with $r_{m}={\left[r_{m}\cos\phi_{m},r_{m}\sin\phi_{m},0\right]}^{T}$ being the position vector of the mth sensor and c being the speed of light [42,43].

#### 2.3.2. MVDR Beamforming Algorithm

We perform Fast Fourier Transform (FFT) on the windowed discrete sampled signals of each array element:

(3) $$\begin{array}{cccc} & X_{m}\left(k,l\right)=\sum_{n=0}^{N-1}x_{m}\left[n+lH\right]h\left[n\right]e^{-j2\pi kn/N} & & \end{array}$$

where N denotes the FFT length, H is the window hop size, $h\left(n\right)$ represents the analysis window function, and k and l denote the frequency bin index and the time frame index, respectively. The corresponding frequency is $f_{k}=kf_{s}/N$.

We then select the desired analysis frequency bin from the frequency domain results for subsequent array localization processing.

For frequency bin k, it corresponding array receiving vector is

$$x_{k}\left(l\right)={\left[X_{1},\left(k,l\right),\ldots X_{M}\left(k,l\right)\right]}^{T}$$

The covariance matrix can be estimated as

(4) $$\begin{array}{cccc} & R_{k}=\frac{1}{L}\sum_{l=1}^{L}x_{k}\left(l\right)x_{k}^{H}\left(l\right) & & \end{array}$$

To minimize array output power while maintaining distortionless response along the target direction, the MVDR beamformer determines the optimal weight vector $\omega_{0,k}$ via constrained optimization:

(5) $$\begin{array}{cccc} & \omega_{0,k}=\frac{R_{k}^{-1}s\left(\theta_{0},\varphi_{0},f_{k}\right)}{s^{H}\left(\theta_{0},\varphi_{0},f_{k}\right)R_{k}^{-1}s\left(\theta_{0},\varphi_{0},f_{k}\right)} & & \end{array}$$

where $s\left(\theta_{0},\varphi_{0},f_{k}\right)$ is the guidance vector of the reference direction, and $\omega_{0,k}$ is the MVDR weight vector of the corresponding frequency bin.

Defining the spatial filtering amplitude response as the ratio of output to input signal amplitude,

(6) $$\begin{array}{cccc} & F_{k}\left(\theta_{d},\varphi_{d}\right)=|\omega_{0,k}^{H}a\left(\theta_{d},\varphi_{d},f_{k}\right)| & & \end{array}$$

where $a\left(\theta_{d},\varphi_{d},f_{k}\right)$ is the array guidance vector in the scanning direction; the normalized beam pattern in decibels is calculated as

(7) $$\begin{array}{cccc} f_{k}\left(\theta_{d},\varphi_{d}\right)=20{log}_{10}\left[\frac{F_{k}\left(\theta_{d},\varphi_{d}\right)}{\underset{\theta_{d},\varphi_{d}}{max}F_{k}\left(\theta_{d},\varphi_{d}\right)}\right] & & & \end{array}$$

where the arrival angles $\left(\theta_{d},\varphi_{d}\right)$ of the radiation source correspond to the angular coordinates at which $f_{k}\left(\theta_{d},\varphi_{d}\right)$ achieves its global maximum. In the numerical simulation, these angular estimates are extracted by conducting a peak search across the normalized beam pattern at the specified single-frequency bin.

In practical signal processing, individual frequency bins can be processed independently, or multiple frequency bins can be jointly analyzed, depending on the specific requirements. Subsequently, methods such as least-squares estimation or geometric averaging can be employed to combine the frequency-dependent beamforming responses and determine the direction of arrival of the lightning radiation source.

## 3. Results

### 3.1. Simulation and Analysis of the Localization and Identification of Two Lightning Radiation Sources

**Figure 2:**
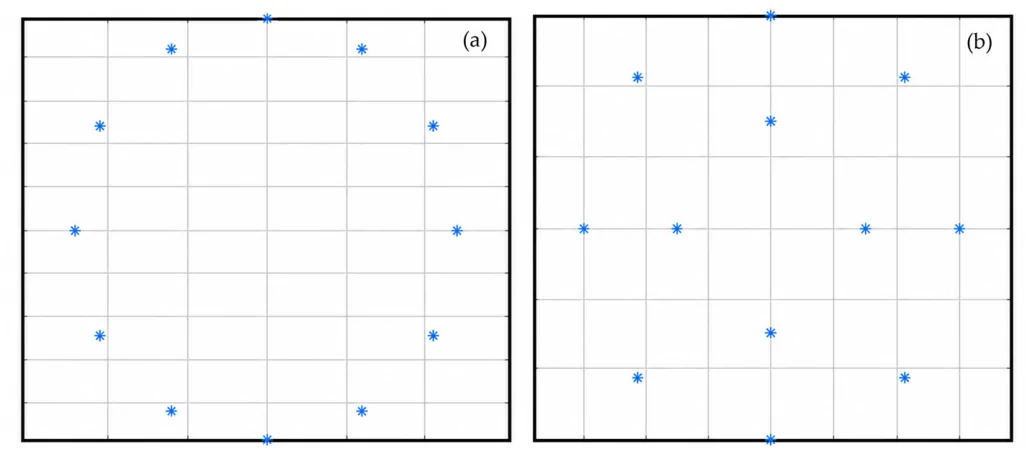
The element distribution structure of the circular planar antenna array used in this article, Each “∗” indicates the position of an array element. (**a**) UCA. (**b**) CCA.

In our previous work [47,48], the theoretical derivations of the MVDR beamforming algorithm and the mathematical model of the Doppler effect in lightning radiation and localization were elaborated in detail. Furthermore, simulation comparisons for the localization of a single stationary lightning source and a single moving lightning radiation source were conducted using a 12-element uniform circular array (UCA) and a 12-element concentric circular array (CCA), respectively. The distribution of array elements is shown in Figure 2, where the coordinate origin is set at the center of the circle, the X-axis is along the horizontal direction, and the Y-axis is along the vertical direction. The results indicated that, under the same number of array elements, using a CCA can better capture the differences in the influence of the Doppler effect on lightning radiation localization compared to using a UCA. Therefore, in this paper, CCA is primarily used to study the influence of the Doppler effect on lightning radiation source localization, while UCA is used to study the localization of multiple lightning radiation sources.

In addition, lightning channels usually exhibit branching during their development, causing multiple radiation sources to appear concurrently. The simplest two-source case is first investigated considering these physical realities in this study. The research strategy is divided into two steps: First, based on previous research on single stationary radiation source localization, the localization simulation for two stationary radiation sources is implemented. Subsequently, by incorporating the motion characteristics of lightning radiation sources—namely the Doppler effect—the localization and identification of two moving lightning radiation sources are achieved. On this basis, the impact of the Doppler effect on the localization of two moving lightning radiation sources is investigated in Section 4 of this paper.

In the localization simulations of this paper, the MVDR beamforming localization algorithm is consistently employed under far-field conditions, where the source-to-element distance satisfies $r>{\left(2D\right)}^{2}/\lambda$, with λ being the signal wavelength and *D* being the array aperture. The radiation frequency of the two lightning radiation sources is set to $1\times 10^{8}$ Hz when stationary, and the radiation frequency range of the moving lightning radiation sources is set within the VHF band of 30–300 MHz. In addition, since lightning electromagnetic radiation is broadband, meaning that it spans a wide frequency spectrum rather than being confined to a single frequency or narrow band, and its dominant frequency varies during different stages of lightning development, the radiation frequencies of the two radiation sources were selected as simulation parameters. A relatively large frequency difference was intentionally chosen to highlight the differences in localization results and facilitate the analysis of frequency-dependent effects.

#### 3.1.1. Localization and Identification of Two Stationary Sources

- Localization of Two Stationary Sources

In the Matlab R2019b implementation of the beamforming localization algorithm, parameters such as the number of array elements, the frequency of the lightning radiation sources, the propagation velocity of the lightning waves, the number of Fourier transform points, and the angles of the lightning radiation source signals are configured first. Subsequently, the sensor array parameters are defined to construct the specific array geometry suitable for lightning radiation source localization. Because the signals radiated by lightning radiation sources possess broadband characteristics, the program performs a Fourier transform on the collected broadband signals, selects the appropriate frequency bins, applies the beamforming algorithm to localize the radiation source, and plots the localization results.

**Figure 3:**
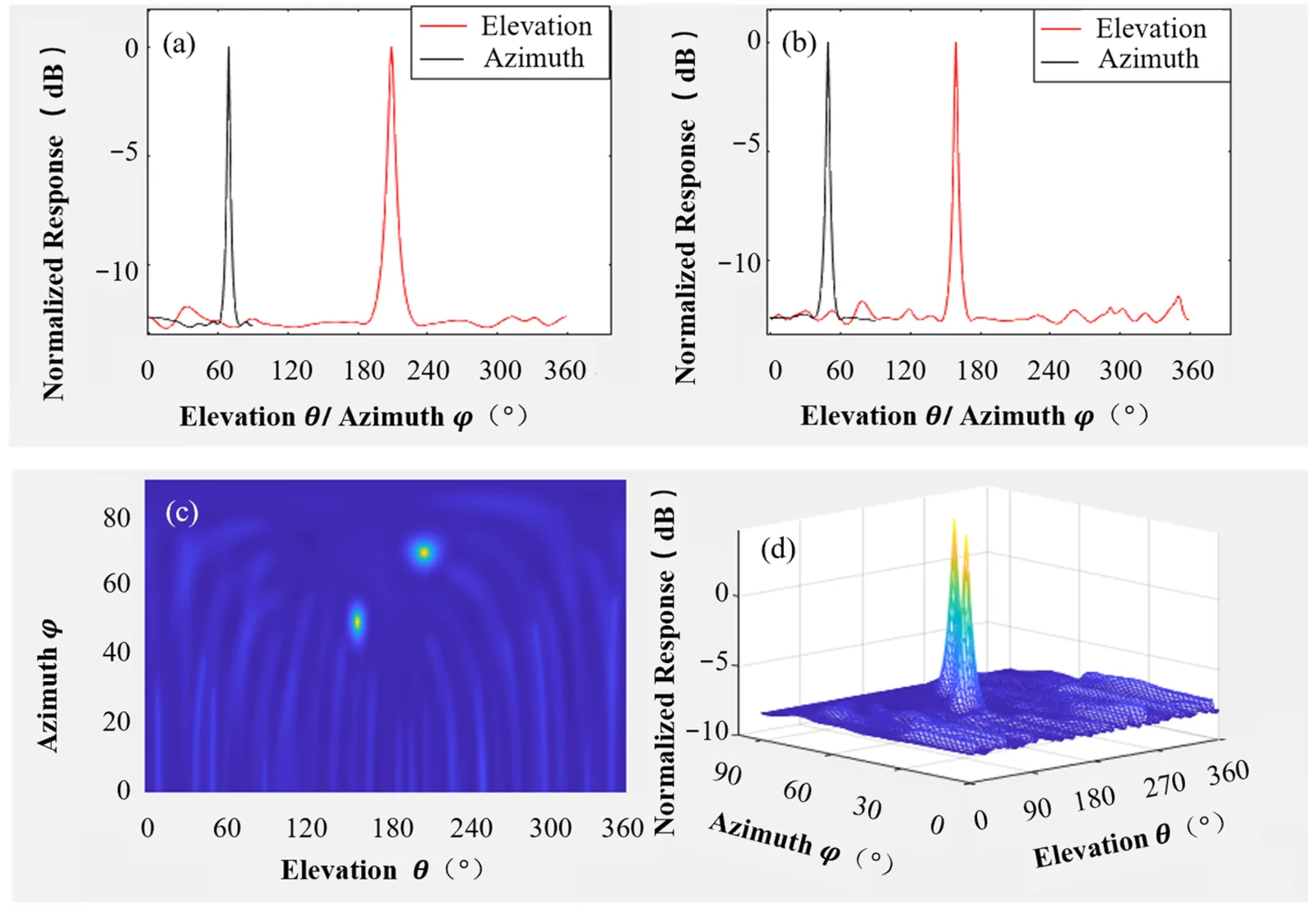
Normalized beam patterns of the MVDR algorithm for two stationary radiation source localizations. (**a**) Normalized azimuth and elevation beam responses for the first radiation source. (**b**) Normalized azimuth and elevation beam responses for the second radiation source. (**c**) 2D normalized beam pattern. (**d**) 3D normalized beam pattern.

During the localization simulation of stationary radiation sources, the radiation frequency of the lightning radiation sources is selected as $1\times 10^{8}$ Hz. Two lightning radiation sources from different directions are randomly selected: the first radiation source has an elevation angle θ of 70° and an azimuth angle φ of 210°; the second radiation source has an elevation angle θ of $50^{0}$ and an azimuth angle φ of 160°. The simulation results are shown in Figure 3: subfigure (a) represents the normalized azimuth and elevation beam responses for the first radiation source, which are the cut profiles of the first radiation source at the corresponding peak of its 3D normalized beam pattern; subfigure (b) represents the normalized azimuth and elevation beam responses for the second radiation source, which are the cut profiles of the second radiation source at the corresponding peak of its 3D normalized beam pattern; subfigure (c) is the 2D normalized beam pattern; and subfigure (d) is the 3D normalized beam pattern.

As shown in Figure 3, the peaks of the beam response curves are quite sharp, the bright spots in Figure 3c are small and concentrated, and the 3D peaks are sharp with excellent directivity. These results indicate that the simulation program can effectively localize two simultaneously occurring radiation sources.

- Identification of Two Stationary Sources

**Figure 4:**
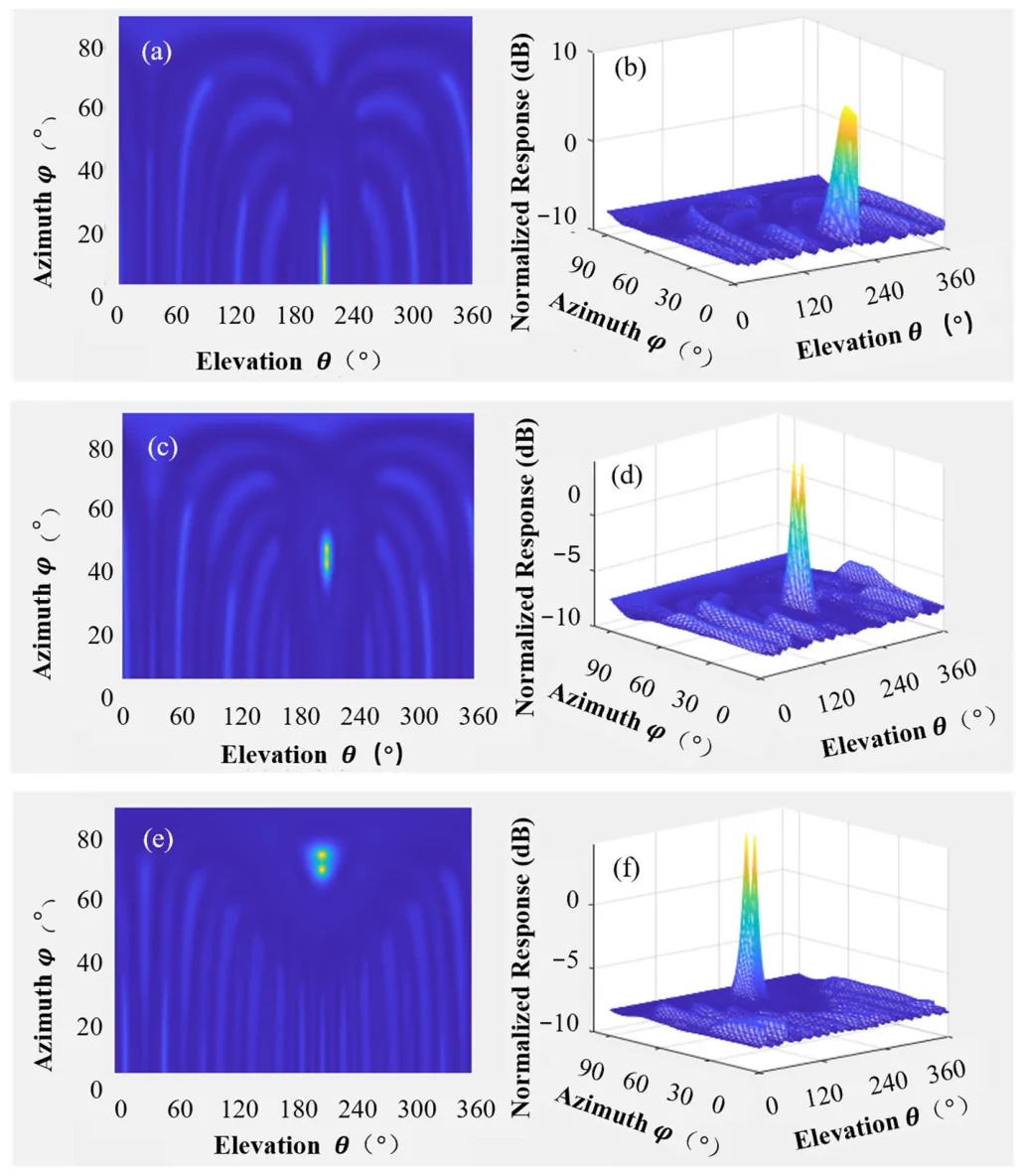
Recognition effect of two stationary radiation sources at different elevation angles. Subfigures (**a**,**b**) correspond to the resolution effect of the positioning system when $\varphi_{1}=\varphi_{2}=210{}^{\circ},\theta_{1}=5{}^{\circ},\theta_{2}=10{}^{\circ}$. Subfigures (**c**,**d**) correspond to the resolution effect of the positioning system when $\varphi_{1}=\varphi_{2}=210{}^{\circ},\theta_{1}=40{}^{\circ},\theta_{2}=45{}^{\circ}$. Subfigures (**e**,**f**) correspond to the resolution effect of the positioning system when $\varphi_{1}=\varphi_{2}=210{}^{\circ},\theta_{1}=70{}^{\circ},\theta_{2}=75{}^{\circ}$. Subfigures (**a**,**c**,**e**) are corresponding 2D normalized beam patterns, while subfigures (**b**,**d**,**f**) are corresponding 3D normalized beam patterns.

A 12-element UCA is constructed using Matlab software to observe the resolution performance of the localization for two stationary radiation sources at different elevation angles. Since it is impractical to display every angle, three sets of data are selected to present the simulation results, as shown in Figure 4.

When the two lightning radiation sources are stationary, the resolution performance of the system varies depending on the elevation angle. As shown in Figure 4a, when the azimuth angles are both 210° and the elevation angles of the two lightning radiation sources are 5° and 10°, respectively, the two radiation sources cannot be accurately distinguished. This further validates the results in Figure 1b, confirming that the spatial resolution is poor within the elevation angle range of 0° to 10°. Conversely, as shown in Figure 4b,c, when the azimuth angles are both 210° and the elevation angles of the two radiation sources are 40°, 45° and 70°, 75°, respectively, the two radiation sources can be accurately resolved. Furthermore, a comparison reveals that the spatial resolution improves as the elevation angle increases, with the resolution in Figure 4c being clearer than that in Figure 4b.

#### 3.1.2. Localization and Identification of Two Moving Sources

- Localization of Two Moving Sources

To investigate the impact of the Doppler effect on the localization results of two moving lightning radiation sources, in this section, the kinematic characteristics of lightning radiation sources are incorporated into the program based on the framework established in the previous section, thereby implementing a simulation system that more closely aligns with practical scenarios. In terms of signal processing, the Doppler effect of broadband signals radiated by the lightning discharge channel is primarily handled via the Fourier transform. By transforming the received time-domain broadband signal into the frequency domain, it is decomposed into discrete frequency bins. For each independent frequency bin, the corresponding Doppler shift is quantitatively evaluated to accomplish frequency-by-frequency phase compensation. Following this Doppler correction, beamforming and localization calculations are then implemented across each frequency component.

**Figure 5:**
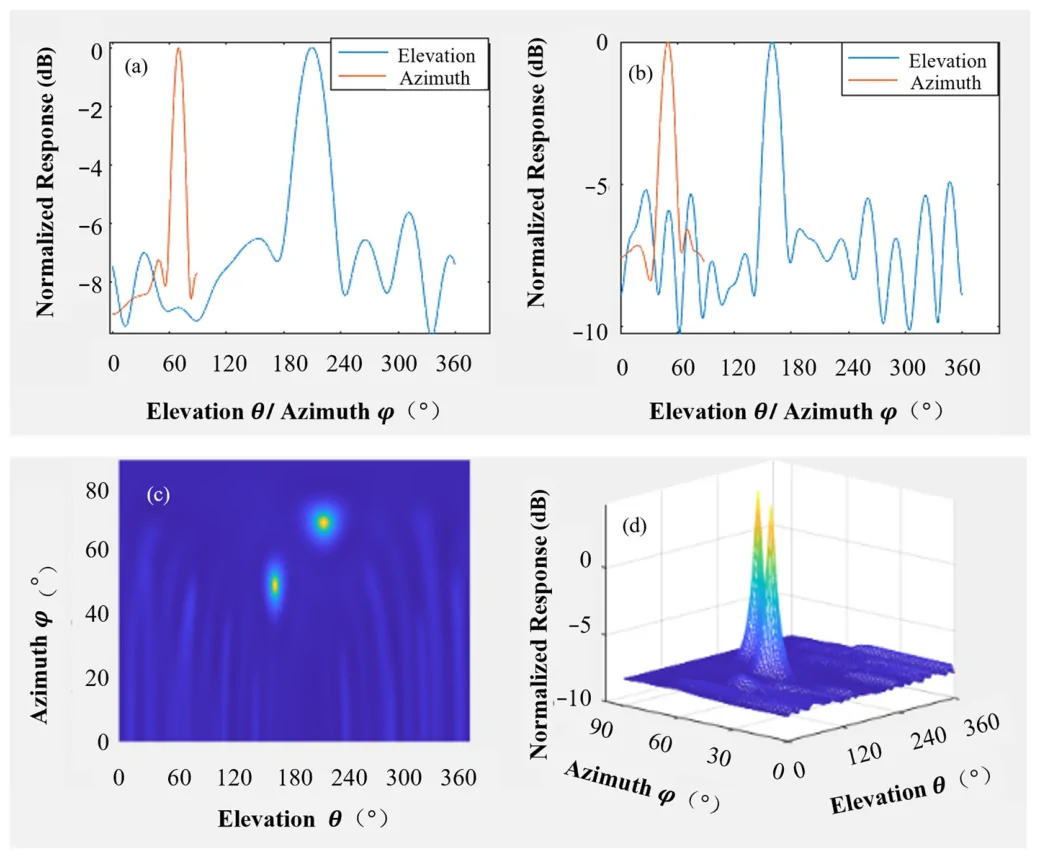
Normalized beam patterns of the MVDR algorithm for two moving radiation source localizations. (**a**) Normalized azimuth and elevation beam responses for the first radiation source. (**b**) Normalized azimuth and elevation beam responses for the second radiation source. (**c**) 2D normalized beam pattern. (**d**) 3D normalized beam pattern.

Taking lightning radiation sources as the research object, since the lightning development process comprises different stages, a distinct dominant frequency of radiation is different in each stage. The dominant frequencies of the two moving lightning radiation sources are specified to maintain consistency with the simulation conditions in the previous section; that is, the radiated main frequencies of both lightning radiation sources are set to $1\times 10^{8}$ Hz. Combined with the MVDR beamforming algorithm, the localization and identification of the lightning radiation sources considering the Doppler effect are performed, and the simulation results are shown in Figure 5.

Comparing Figure 3 with Figure 5, due to the influence of the Doppler effect, the beam response curves for the elevation and azimuth angles of the two lightning radiation sources in Figure 5a,b become smoother, and their main-lobe widths widen, leading to degraded localization performance. In Figure 5c,d, the bright spots in the 2D normalized beam pattern expand significantly and become divergent, and the directivity in the 3D normalized beam pattern in Figure 5d decreases markedly. Under identical conditions, the localization accuracy will also decrease due to the influence of the Doppler effect.

- Identification of Two Moving Sources

**Figure 6:**
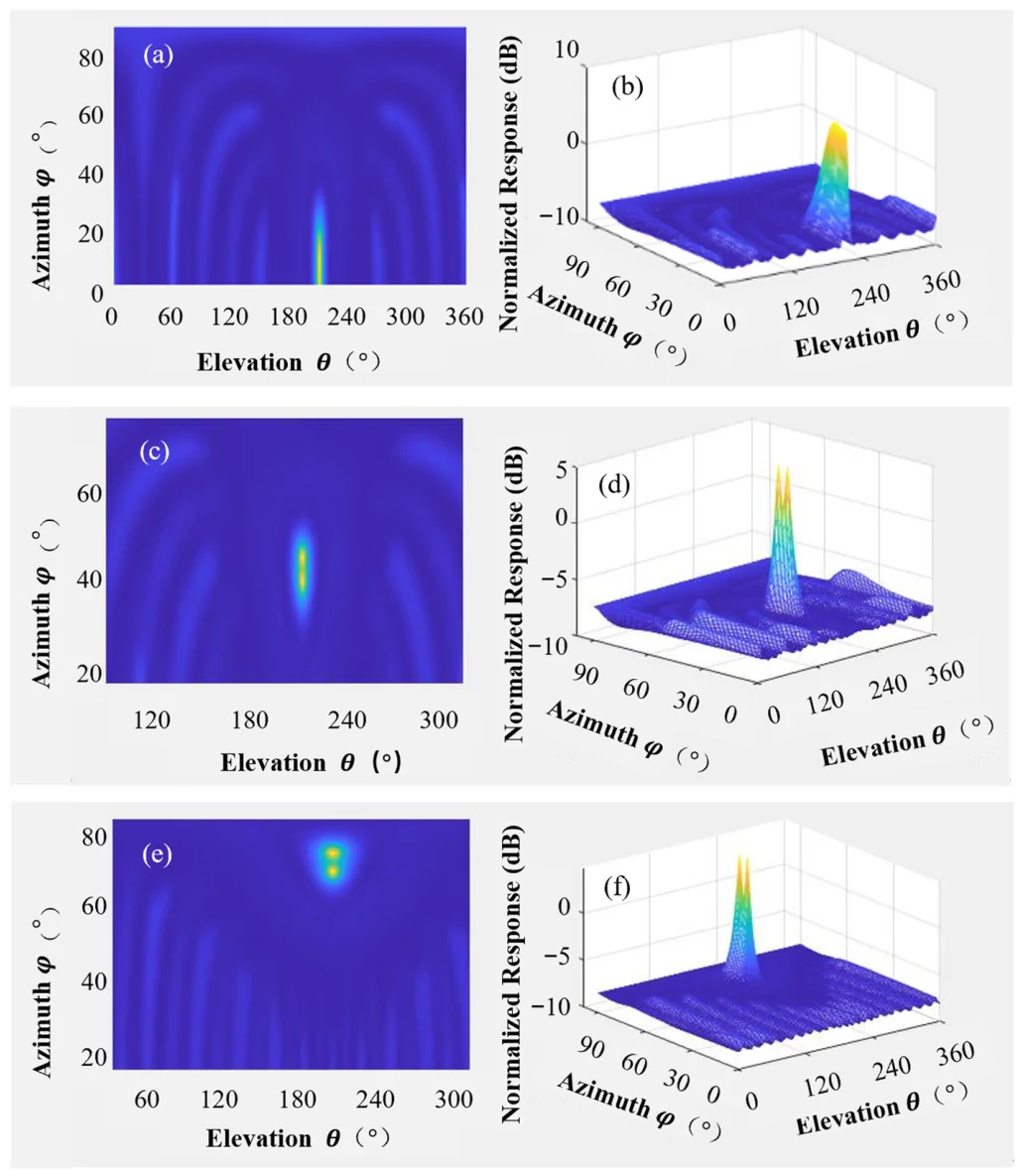
Recognition effect of two moving radiation sources at different elevation angles. Subfigures (**a**,**b**) correspond to the resolution effect of the positioning system when $\varphi_{1}=\varphi_{2}=210{}^{\circ},\theta_{1}=5{}^{\circ},\theta_{2}=10{}^{\circ}$. Subfigures (**c**,**d**) correspond to the resolution effect of the positioning system when $\varphi_{1}=\varphi_{2}=210{}^{\circ},\theta_{1}=40{}^{\circ},\theta_{2}=45{}^{\circ}$. Subfigures (**e**,**f**) correspond to the resolution effect of the positioning system when $\varphi_{1}=\varphi_{2}=210{}^{\circ},\theta_{1}=70{}^{\circ},\theta_{2}=75{}^{\circ}$. Subfigures (**a**,**c**,**e**) are corresponding 2D normalized beam patterns, while subfigures (**b**,**d**,**f**) are corresponding 3D normalized beam patterns.

Following the same simulation procedure as described above for two stationary radiation sources, a 12-element UCA is constructed in Matlab software. Then, motion characteristics are incorporated, and the resolvability of the localization results for two moving radiation sources is observed. Three sets of data are likewise selected to display the simulation results. As shown in Figure 6a, when the azimuth angles are both 210° and the elevation angles of the two lightning radiation sources are 5° and 10°, respectively, the two radiation sources cannot be accurately distinguished. As shown in Figure 6b,c, when the azimuth angles are both 210° and the elevation angles are 40°, 45° and 70°, 75°, respectively, the two radiation sources can be accurately distinguished.

A comparison indicates that the spatial resolution improves as the elevation angle increases, which is consistent with the simulation results obtained for the two stationary radiation sources. In practical localization, for lightning at different distances, when the circular planar antenna array position remains fixed and the cloud height is the same, the farther the two lightning radiation sources are from the receiving array, the smaller the elevation angle, and the spatial resolution will be poorer. Conversely, the closer the two lightning radiation sources are to the circular planar antenna array, the larger the elevation angle, and the spatial resolution will be better. When two lightning strikes simultaneously occur from clouds at different heights, their elevation angles differ in magnitude. The higher the cloud layer from which two simultaneously occurring lightning radiation sources originate, the larger the elevation angle and the better the spatial resolution; conversely, the spatial resolution will degrade.

### 3.2. The Influence of Source Radiation Frequency on the Localization and Identification of Moving Sources

When multiple lightning events occur simultaneously, they may be in different discharge stages, causing different lightning radiation sources to radiate electromagnetic waves with different dominant frequencies at the same time. Conversely, when a single lightning flash exhibits multiple branches or grounding points, the various branches may be in identical or similar discharge stages, leading to identical or close radiation main frequencies among the different lightning radiation sources. The impact of the Doppler effect on the localization of two moving lightning radiation sources will be analyzed in this section. Specifically, the localization and identification results under the influence of the Doppler effect are comparatively investigated for two scenarios: two lightning radiation sources with identical radiation main frequencies and two lightning radiation sources with different radiation main frequencies.

#### 3.2.1. For Two Sources with Identical Radiation Main Frequencies

Considering the practical scenarios that may occur during lightning events, the influence of the Doppler effect on the localization results of two moving lightning radiation sources was further investigated in this section. The discharge process between a thundercloud and the ground is not a single event. During a lightning discharge process, the most common form of lightning is linear discharge, with linear lightning resembling an inverted, branching tree branch. As lightning develops from the cloud toward the ground, the stepped leader frequently moves along tortuous paths in irregular spatial patterns, thereby forming a zigzagging main channel that radiates numerous branches into the surroundings.

**Figure 7:**
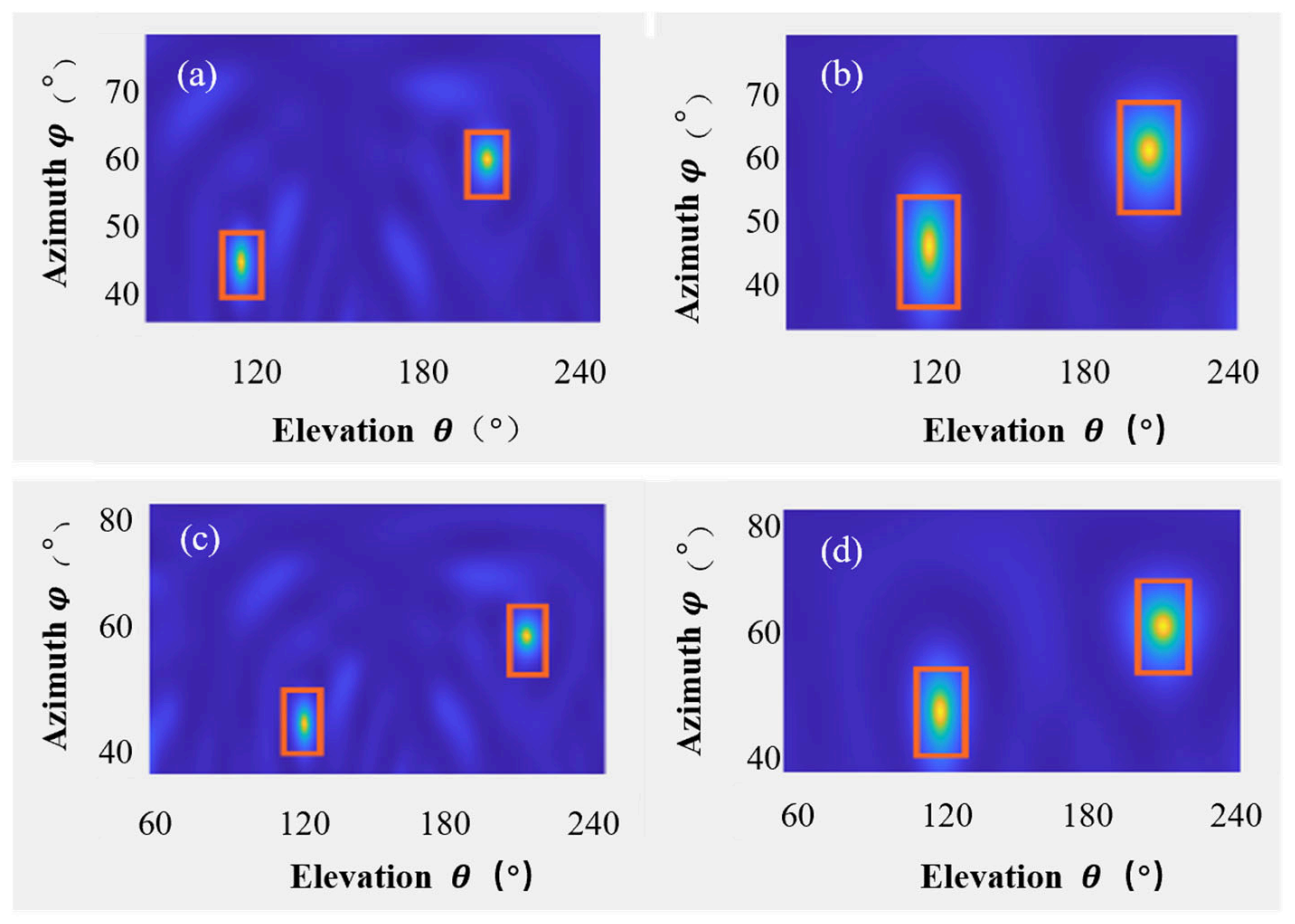
Comparison of the effects of the Doppler effect on the localization results of two same-frequency radiation sources at different frequencies. Subfigures (**a**,**b**) correspond to the case where the frequency is $f_{s_{1}}=f_{s_{2}}=3\times 10^{7}\;Hz$. Subfigures (**c**,**d**) correspond to the case where the frequency is $f_{s_{1}}=f_{s_{2}}=3\times 10^{8}\;Hz$. Subfigures (**a**,**c**) are the cases without considering the Doppler effect, while subfigures (**b**,**d**) are the cases considering the Doppler effect.

Assuming that two lightning radiation sources radiate at the same dominant frequency, here, the scenario where the dominant frequency of lightning radiation falls within the VHF band is primarily investigated. Assuming that the incoming wave directions (elevation angle, azimuth angle) are (60°, 210°) and (45°, 120°), respectively, and keeping all other parameters constant, the radiation main frequencies of the two lightning radiation sources are varied from $3\times 10^{7}$ Hz to $3\times 10^{8}$ Hz with a fixed step size for localization simulations. Two representative frequencies, $3\times 10^{7}$ Hz and $3\times 10^{8}$ Hz, are selected for presentation and analysis (as shown in Figure 7) to compare the localization performance with and without considering the Doppler effect.

From Figure 7, it can be observed that when the Doppler effect is not considered, the high-intensity spot regions in the 2D normalized beam pattern of the two lightning radiation sources are small, regular, and concentrated, exhibiting superior directivity and yielding higher spatial accuracy in the localization results. Conversely, when the Doppler effect is taken into account, the spots in the 2D normalized beam pattern for both lightning radiation sources become big and visually dispersed and show poorer directivity, thereby degrading the system’s overall localization performance. Consequently, during the progression of lightning, especially when branching occurs or two lightning channels develop simultaneously, the Doppler effect simultaneously affects the localization results for both lightning radiation sources. Furthermore, this impact intensifies as the radiation frequency of the lightning radiation source increases, leading to larger errors and greater deviation between the estimated localization results and the actual positions.

#### 3.2.2. Two Lightning Sources with Different Frequencies

In contrast to the identical-frequency scenario, during a lightning event, branching frequently occurs, or two flashes may occur simultaneously. In such cases, different lightning development stages may correspond to different dominant frequencies. Therefore, the impact on localization simulations is further investigated for scenarios where the two lightning radiation sources have different frequencies.

**Figure 8:**
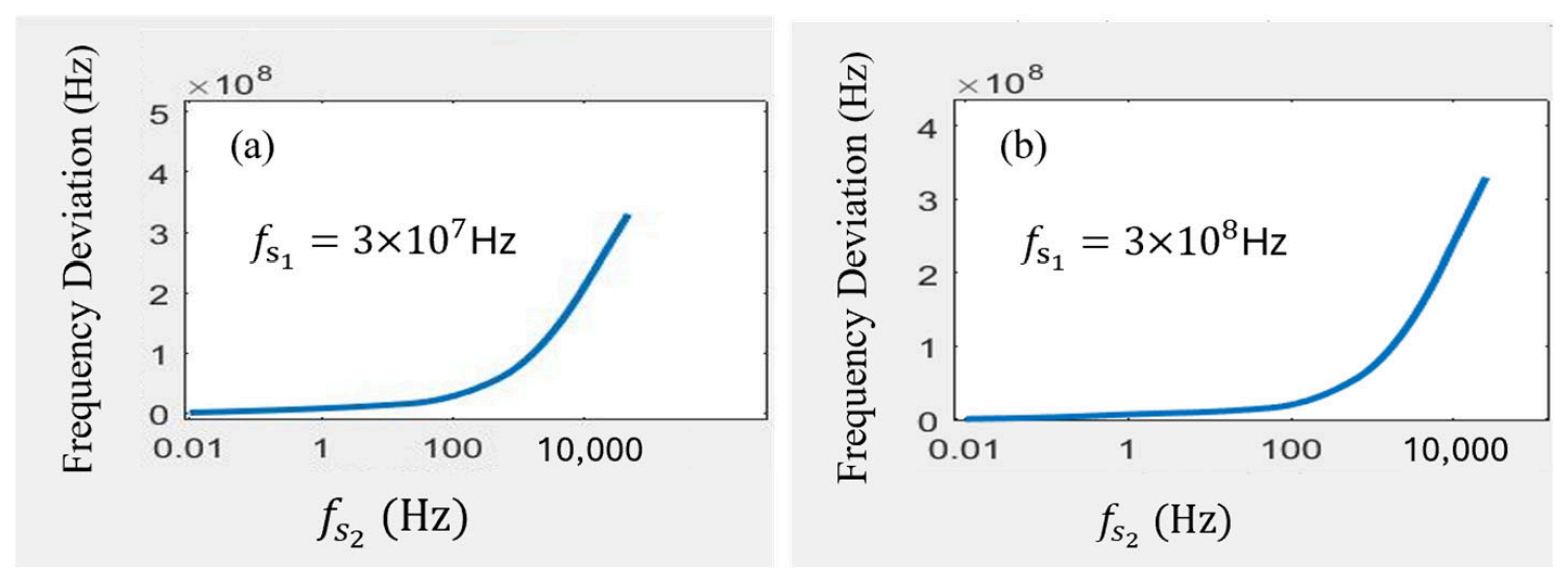
The frequency deviation curve of two moving radiation sources with different frequencies affected by Doppler effect. Subfigure (**a**) shows the frequency deviation between the radiation frequency of radiation source 2 and the frequency of its signal received by the circular planar antenna array as a function of the radiation frequency of radiation source 2, with the frequency of lightning radiation source 1 fixed at $3\times 10^{7}$ Hz. Subfigure (**b**) shows the frequency deviation between the radiation frequency of radiation source 2 and the frequency of its signal received by the circular planar antenna array as a function of the radiation frequency of radiation source 2, with the frequency of lightning radiation source 1 fixed at $3\times 10^{8}$ Hz.

To compare the localization results and ascertain the underlying variation patterns, two sets of simulation conditions are selected. In both simulation procedures, the radiation main frequency of the first lightning radiation source ($f_{s_{1}}$) is kept constant within the VHF band, specifically set to $3\times 10^{7}$ Hz and $1\times 10^{8}$ Hz for the two respective simulation runs. Meanwhile, the radiation main frequency of the second lightning radiation source ($f_{s_{2}}$) is varied. To capture a comprehensive variation pattern, the frequency range of $f_{s_{2}}$ is chosen to be wider than the standard VHF band, spanning from $1\times 10^{4}\;Hz\;to\;1\times 10^{11}$ Hz. Simulations are performed according to these criteria, and the results are presented in Figure 8a,b. Figure 8a,b illustrate the variation in the Doppler-induced frequency deviation between the radiation source and received frequencies of lightning radiation source 2 with $f_{s_{2}}$, while the frequency of lightning radiation source 1 is maintained at $3\times 10^{7}$ Hz and $1\times 10^{8}$ Hz, respectively.

An analysis of Figure 8 reveals that the error value increases as $f_{s_{2}}$ increases. When $f_{s_{2}}$ is much smaller than $f_{s_{1}}$, the frequency error of lightning radiation source 2 induced by the Doppler effect remains relatively small, and the error curve varies smoothly. However, when $f_{s_{2}}$ is much larger than $f_{s_{1}}$, the discrepancy between the orders of magnitude of the two frequencies widens; consequently, the frequency error curve of lightning radiation source 2 changes more rapidly, asymptotically approaching a straight upward trend.

**Figure 9:**
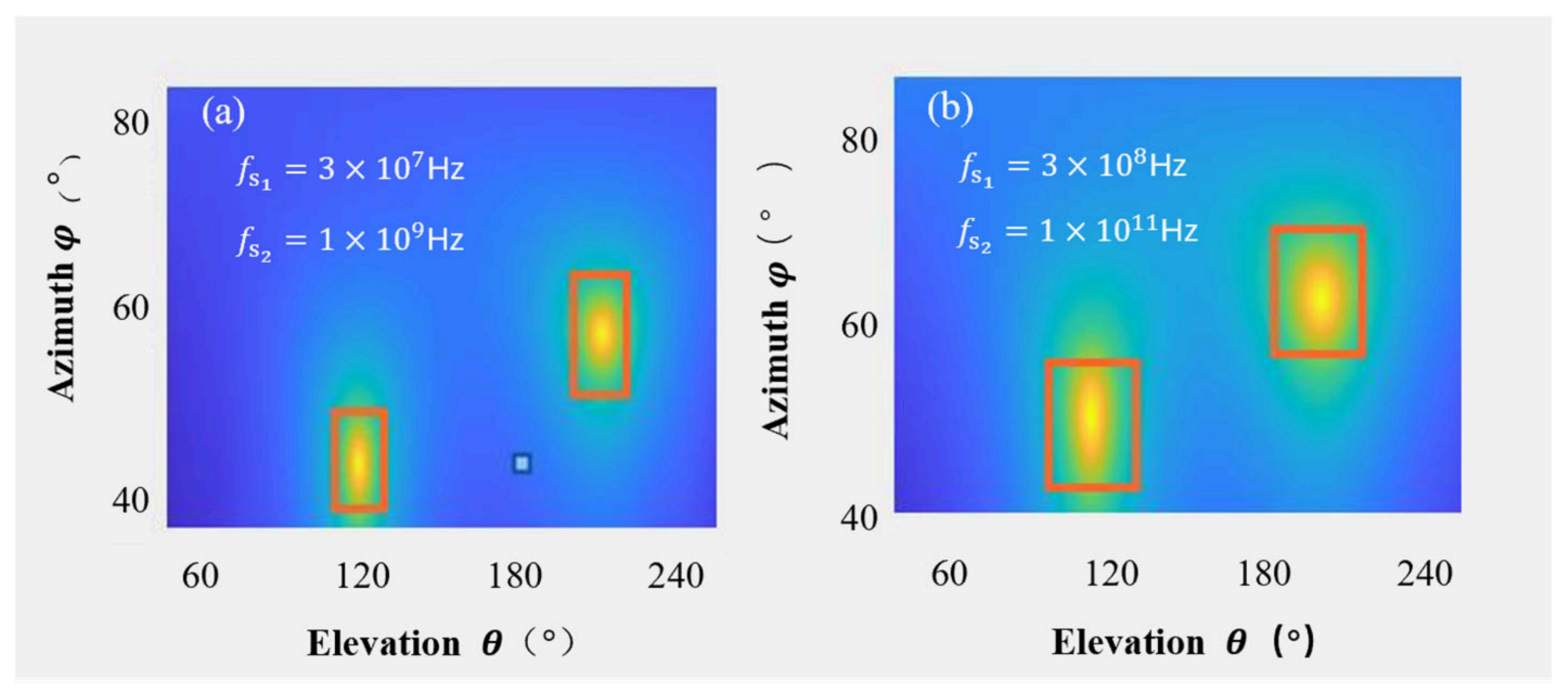
Comparison of 2D normalized beam pattern of two radiation sources with different radiation frequencies. Subfigure (**a**) corresponds to the situation of $f_{s_{1}}=3\times 10^{7}$ Hz and $f_{s_{2}}=1\times 10^{9}$ Hz, while subfigure (**b**) corresponds to the situation of $f_{s_{1}}=3\times 10^{8}$ Hz and $f_{s_{2}}=1\times 10^{11}$ Hz.

To provide a clearer and more intuitive comparison of the influence of the Doppler effect on the localization of two radiation sources with different radiation frequencies, two sets of data are selected to display the localization simulation results. The frequency range of radiation source 2 is the same as that used in Figure 8. The first group compares the case where the frequencies of the two radiation sources are $f_{s_{1}}=3\times 10^{7}$ Hz and $f_{s_{2}}=1\times 10^{9}$ Hz, while the second group uses $f_{s_{1}}=3\times 10^{8}$ Hz and $f_{s_{2}}=1\times 10^{11}$ Hz, as shown in Figure 9.

As shown in Figure 9a, when the frequencies of the two lightning radiation sources differ by two orders of magnitude, the bright spot region in the 2D normalized beam pattern is relatively small and concentrated, indicating better directivity and relatively high localization accuracy. As shown in Figure 9b, when the frequencies of the two lightning radiation sources differ by three orders of magnitude, the bright spot region in the 2D normalized beam pattern is larger and more dispersed, resulting in poorer directivity and reduced localization accuracy. A comparison of Figure 9a,b shows that, as the frequency difference between the two lightning radiation sources increases, the influence of the Doppler effect becomes more pronounced, leading to a larger localization error. Conversely, a smaller frequency difference results in a smaller localization error. Evidentially, these simulation results are in agreement with the variation trends shown in Figure 8.

## 4. Discussion

The simulation results of this study demonstrate that the localization accuracy of the circular planar array is closely tied to the spatial resolution of the system and significantly depends on the spatial geometric position of the radiation sources relative to the receiving circular planar array. Notably, the spatial resolution exhibits a dramatic increase at an elevation angle of $\theta =10^{0}$; meanwhile, higher cloud altitudes (i.e., larger elevation angles) yield superior spatial resolution. This is consistent with the fundamental understanding in array signal processing theory that spatial resolution is governed by both the direction of arrival and the array geometry, which also validates the research hypothesis of this paper that the spatial position and motion characteristics of lightning radiation sources affect the localization results. From a physical mechanism perspective, a larger elevation angle provides a more favorable aperture projection and a steeper phase difference gradient, thereby significantly enhancing the algorithm’s super-resolution capability to distinguish spatially adjacent dual radiation sources. This indicates that, in practical lightning observation, reasonable array geometric layout and observation angle selection are crucial for capturing concurrent multiple lightning channels and complex multi-branching lightning events. The potential use of non-coplanar antenna configurations to improve localization performance at low elevation angles is identified as a direction for future research [17].

Although the theoretical spatial resolution expression is based on an idealized planar aperture model, both the UCA and CCA used in the simulations are planar array configurations. This theoretical expression provides a reference for understanding the dependence of spatial resolution on circular planar antenna array aperture, wavelength, and elevation angle, rather than an exact characterization of the resolution performance of specific array geometries. Since the actual resolution also depends on the array geometry, MVDR-based numerical simulations are employed to further evaluate the spatial resolution performance of UCA and CCA.

Meanwhile, this study extends the stationary radiation source identification theory to dynamic moving radiation sources, systematically revealing the mechanism by which the Doppler effect acts on localization errors. The results show that localization errors increase significantly with higher source radiation frequencies and larger frequency differences between the dual radiation sources. In previous lightning radiation source localization studies, short-duration radiation sources were typically simplified as stationary point sources, neglecting the frequency shifts induced by the high-speed propagation of leaders or return strokes (with speeds reaching $10^{7}\sim 10^{8}\;m/s$) along the channel. In this study, it is confirmed that under high-frequency bands and multi-frequency concurrent scenarios, the Doppler shift induces an additional array phase offset in the received signals, ultimately leading to a systematic shift in the localization peaks. This finding suggests that, in high-precision research on lightning microphysical processes, it is necessary to incorporate radiation source motion and its frequency shift characteristics into the localization model and introduce corresponding Doppler effect compensation mechanisms.

It should be noted that the localization and identification features of dual lightning radiation sources are focused in this study. In actual ground flashes, however, concurrent multiple branches or simultaneous lightning flashes often result in three or more radiation sources entering the same observation window. Compared with the dual-source scenario, multi-source localization is jointly constrained by multiple factors, including the number of array elements, angular spacing between sources, signal correlation, and signal-to-noise ratio. As the number of concurrent radiation sources increases, the spatial spectral peaks of different sources may become closer under finite array aperture conditions; particularly in low-elevation-angle regions, inter-source resolvability may decline further, increasing the likelihood of spectral peak overlapping or artifact generation. Furthermore, for multiple moving radiation sources, variations in motion direction, propagation speed, and radiation frequency among different lightning branches can produce even more complex combinations of frequency shifts, further complicating steering vector mismatches and source-association tasks. Consequently, spatial coupling, frequency differences, and dynamic motion characteristics under multi-source conditions make the localization problem nonlinearly complex, meaning the dual-source conclusions of this study cannot be directly extrapolated to arbitrary multi-source scenarios. Nevertheless, as the baseline for analyzing complex multi-source scenarios, the dual-source model not only reveals the fundamental physical mechanisms governing how spatial geometry and the Doppler effect impact localization, but also serves as an indispensable theoretical foundation and methodological reference for constructing future multi-source localization algorithms and error models.

Overall, analyzing the localization problem of dual lightning radiation sources from the two perspectives of spatial resolution characteristics and source-motion Doppler effects can provide a solid theoretical foundation for subsequent lightning array observations and fine-structure research on lightning channels. Future research will proceed along the following directions. On the one hand, it can integrate actual high-speed camera observation with electromagnetic observation data to investigate the coupling mechanism between array spatial resolution and the Doppler effect under three or more moving lightning radiation sources, thereby establishing a dynamic steering vector correction algorithm that considers the Doppler effect to eliminate motion biases. On the other hand, it can optimize the array topology and explore super-resolution optimization strategies for 3D non-planar arrays in low-elevation-angle regions, and comprehensively evaluate the influence of factors such as source spacing, motion direction, frequency combinations, and signal-to-noise ratios to provide more solid algorithmic support for fine-structure characterization of lightning channels and electromagnetic field observations in complex lightning radiation environments. The present study focuses on the influence of the Doppler effect on localization performance, while the potential effects of adverse weather conditions remain outside its scope and warrant further investigation.

## 5. Conclusions

Based on the localization and identification theory for two lightning radiation sources, the localization of two stationary radiation sources is investigated in this paper. On this basis, the kinematic characteristics of the lightning radiation sources are incorporated—namely, accounting for the Doppler effect—to achieve the localization of two moving lightning radiation sources. Finally, by comparing the localization performance with and without considering the Doppler effect, the impact of the Doppler effect on the localization results of two moving lightning radiation sources is investigated. The main conclusions are as follows:

1. For two lightning radiation sources, the spatial resolution capability of the planar antenna array localization system regarding the lightning radiation sources depends on the elevation angle θ of the radiation sources. As θ increases, the spatial resolution increases. The resolution performance is poor at small elevation angles, particularly within the range of 0–10°. Hence, this range should be avoided as much as possible during actual observation.
2. For two lightning radiation sources with identical radiation main frequencies, the impact of the Doppler effect on the localization results of the two moving radiation sources intensifies as the lightning radiation source frequency increases. Consequently, the induced errors enlarge, and the deviation between the localized results and the actual positions widens.
3. For two lightning radiation sources with different radiation main frequencies, when the frequency of one lightning radiation source increases, the impact of the Doppler effect on the error between its source radiation frequency and received frequency increases. A larger discrepancy between the frequencies of the two lightning radiation sources renders the influence of the Doppler effect on the localization results of the two moving radiation sources more pronounced, thereby increasing the errors in the localization results.

In the simulation analysis of this paper, only the localization, identification, and Doppler effect impact regarding two radiation sources are investigated. Scenarios involving multiple sources will be more complex, and further in-depth research on this subject is required during subsequent field observations of lightning. This work establishes a theoretical foundation for refined observations and detailed studies of lightning.

## Data Availability

The data presented in this study are available from the corresponding author upon reasonable request.

## Abbreviations

The following abbreviations are used in this manuscript:

MVDR	Minimum Variance Distortionless Response
UCA	Uniform circular array
CCA	Concentric circular array
3D	Three-dimensional
VHF	Very high frequency
TOA	Time-of-arrival
LDAR	Lightning detection and ranging
LMA	Lightning mapping array
TDOA	Time difference of arrival
DOA	Direction of arrival
2D	Two-dimensional
MUSIC	Multiple signal classification
ESPRIT	Estimation of Signal Parameters via Rotational Invariance Techniques
LOFAR	Low-frequency array
FWHM	Full width at half-maximum
